# Concavities of the margins of focal bone lesions on MRI: a retrospective study of 586 cases

**DOI:** 10.1186/s13244-025-02137-9

**Published:** 2025-11-10

**Authors:** Abdulrazak Kalaaji, Anthony De Leeuw, Simon Henry, Nathalie Boutry, Sammy Badr, Anne Cotten

**Affiliations:** 1 Department of Musculoskeletal Imaging, CIAL, Lille University Hospital, Lille, France; 2 Lille Faculty of Medicine, Lille University, Lille, France; 3 Department of Pediatric Radiology, Jeanne de Flandre Hospital, Lille University, Hospital, Lille, France

**Keywords:** Magnetic resonance imaging, Bone neoplasms, Fibrous dysplasia of bone, Bone necrosis, Infectious bone diseases

## Abstract

**Objectives:**

Except for a few specific lesions, the analysis of the margins of focal bone lesions with MRI has been largely overlooked in the literature. We observed that some lesions exhibited concave margins, suggesting a non-aggressive nature. This study aimed to determine whether concave borders are more frequently present in certain types of focal lesions of the long bones on MRI, particularly in benign lesions.

**Materials and methods:**

MRI examinations of 586 focal intraosseous lesions of the long bones were retrospectively reviewed. Each lesion margin was independently classified by two musculoskeletal radiologists as concave, non-concave, or cortical. The number of concave margins was analyzed according to the lesion type and its classification as tumoral (benign or malignant) or non-tumoral.

**Results:**

The study group included 15 different types of lesions (75.1% tumors, 24.9% non-tumoral lesions). The number of concave margins per lesion varied significantly by lesion type (*p* < 0.001). Benign lesions and benign tumors had more concave margins than malignant lesions (*p* < 0.001). All osteonecrosis lesions, 44.8% of fibrous dysplasia lesions, 20.8% of abscesses, 14.8% of chondroblastomas, 12.5% of Langerhans cell histiocytosis lesions, and 2.9% of metastases showed at least two concave margins. No lesions in the other groups had more than one concave margin.

**Conclusion:**

Our study revealed that several intraosseous lesions tend to exhibit concave margins. The presence of at least two smooth and regular inward-curved margins was most commonly found in benign lesions and benign tumors, such as fibrous dysplasia.

**Critical relevance statement:**

This study shows that at least two smooth, inward-curved margins are more common in benign bone lesions and tumors and may help in recognizing fibrous dysplasia in non-fatty lesions.

**Key Points:**

- Identifying concave margins may help in recognizing benign bone lesions and tumors.
- Concave margins are mainly observed in osteonecrosis and fibrous dysplasia.
- Concave margins suggest a non-aggressive nature of focal bone lesions.

**Graphical Abstract:**

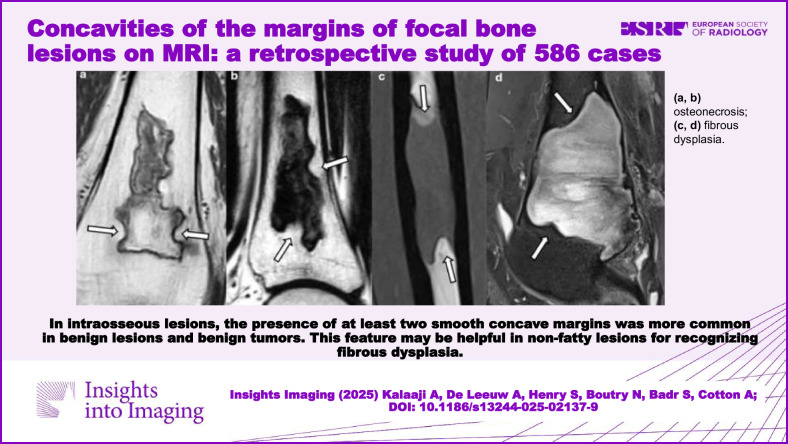

## Introduction

Focal bone lesions are frequently encountered in clinical practice. They present a wide differential diagnosis that includes primary and secondary bone tumors as well as non-tumoral bone lesions. The analysis of these lesions involves both non-radiological features (such as age, medical history, clinical symptoms, and biological markers) and radiological features (including location, size, number of lesions, margins, matrix, adjacent trabecular, cortical, and medullary bone, periosteal reaction, and adjacent soft tissue involvement) [1–4]. One of the primary challenges for radiologists is to identify “do not touch lesions” and, for other lesions, to assess their aggressiveness and, if possible, propose a diagnosis.

The analysis of lesion margins is essential for assessing aggressiveness. This feature is well-described radiographically in the Lodwick-Madewell classification [5]. However, the shape of the lesion, particularly its margins, remains underexplored in the literature, except for specific lesions, such as lobulated margins in cartilaginous tumors [6–8].

In our practice, we observed that certain intraosseous lesions in long bones, when assessed with magnetic resonance imaging (MRI), exhibit focal or extensive concave inward margins. This feature should argue against an expansile or aggressive process, which typically presents convex margins. To the best of our knowledge, this feature has not been previously studied in the literature. Consequently, its prevalence, its association with specific lesion types—particularly benign ones—and its potential usefulness in clinical practice remain unknown. We hypothesized that this novel marker could improve radiological assessment of bone lesions. Therefore, this study aimed to determine whether concave margins are more commonly associated with specific types of focal bone lesions, particularly benign ones.

## Materials and methods

### Study group

This retrospective observational study was conducted at the University Hospital of Lille in conformity with ethical regulations and with approval from the Institution’s Data protection officer (DEC23-145). MRI reports generated in our department from January 2015 to December 2021 were reviewed using an electronic keyword search (Illuminate, Softek). The eligibility criteria for recruitment were (1) focal bone lesions located within the long bones of the appendicular skeleton and (2) MRI examination with at least two orthogonal planes and a maximum slice thickness of 4 millimeters. All MRI examinations were performed using either a 1.5-T or 3-T full-body scanner (Ingenia 1.5 T and Ingenia 3 T; Philips Medical Systems). Images were accessible in the hospital’s picture archiving and communication system (IntelliSpace PACS; Philips Healthcare). Electronic medical records, including demographic and clinical data, were available for each patient.

The total number of eligible lesions was 1076. Of these, 490 were excluded for the following reasons: (1) uncertain diagnosis from pathological, bacteriological, imaging, and/or follow-up data (118 lesions); (2) aggressive osteolysis of the cortex and/or pathological fracture, which impeded margin analysis (131 lesions), (3) history of lesion treatment or presence of implanted material (41 lesions); (4) small lesion size, less than 1 centimeter, preventing accurate margin analysis (133 lesions); and (5) suboptimal MRI quality due to protocol issues, artifacts, or patient movements (128 lesions).

Our final cohort included 586 lesions, each reliably diagnosed by one of four methods: (1) histology (127 lesions) or bacteriology (24 lesions) obtained via core biopsy needle or surgical resection; (2) typical “do not touch” bone lesion appearance, in accordance with established criteria (203 lesions) [9–17]; (3) multidisciplinary consensus, or interpretation by a senior musculoskeletal radiologist (A.C.), combined with at least 2 years of imaging follow-up (232 lesions).

### MRI analysis

Forty MRI examinations from the dataset were initially used for the training. Subsequently, MRI examinations were independently analyzed by two musculoskeletal radiologists with one (A.K.) and more than 20 years (A.C.) of experience in musculoskeletal radiology, respectively. The scans were randomized, and the readers were blinded to the final diagnosis. For intra-observer agreement, the less experienced radiologist reassessed 118 of the 586 lesions three months later.

The following MRI features were recorded for each lesion: the affected long bone; its epiphyseal, metaphyseal, and/or diaphyseal location; the lesion’s size along its long axis; and the type of margin for each of its borders (anterior, posterior, medial, lateral, proximal, and distal).

**Fig. 1:**
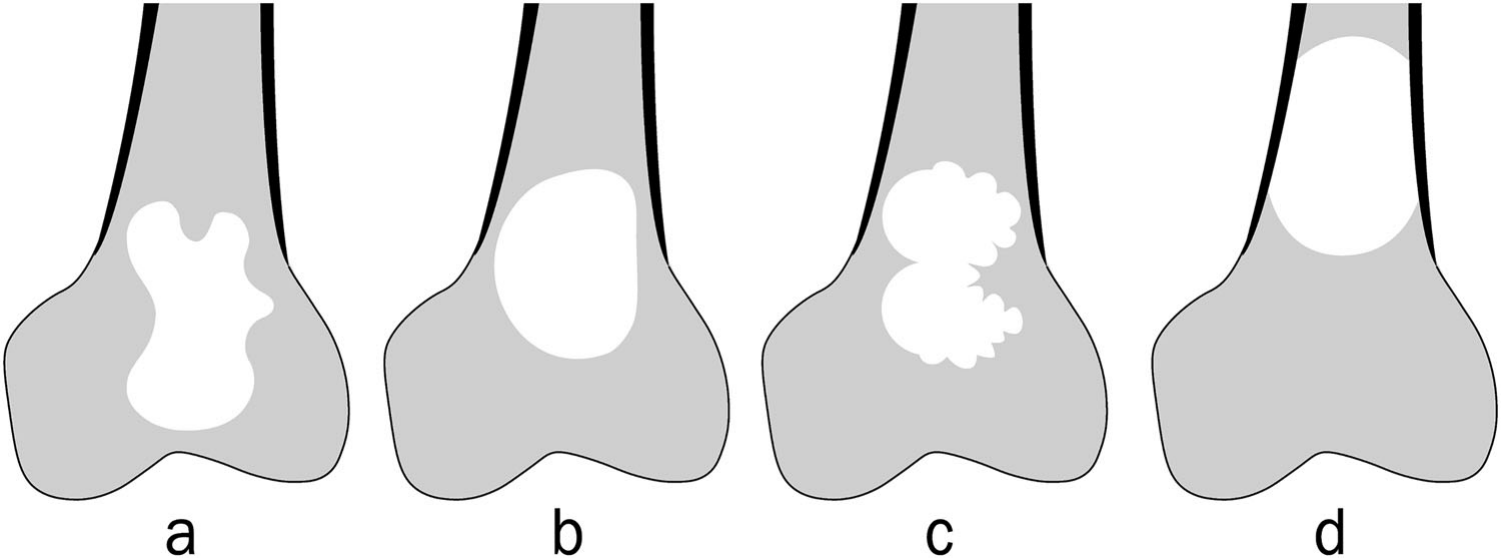
Four examples of bone lesions with (**a**) lateral, medial and distal concave margins (white arrows), (**b**) convex (black arrows) and linear margins, (**c**) pseudo-concavities with a large indentation on the lateral border bordered by two convex borders (star), and pseudo-concavities created by small indentations on the rest of the borders, and (**d**) cortical medial and lateral margins

Each margin was classified and defined as follows (Fig. 1): (1) concave: at least one focal concavity or a complete concave margin, defined by a smooth and regular inward-curved contour; (2) not concave: the absence of concavity in the lesion margin, as defined above. This group included convex or linear borders, and pseudo-concavities such as small or large indentations bordered by two convex borders; and (3) cortical: when the lesion margin was predominantly in contact with the cortex, preventing a reliable analysis of the margin.

### Statistical analysis

The Kruskal–Wallis test was used to determine if there was a statistically significant difference in the number of concave margins per lesion among the groups studied, followed by a post hoc multiple pairwise comparison analysis using the Dunn test with Bonferroni correction. The Wilcoxon–Mann–Whitney test was conducted to assess significant differences in the number of concave margins per lesion between non-tumoral and tumoral lesions, between benign lesions (non-tumoral and benign tumoral lesions) and intermediate/malignant tumors, and between benign tumors and intermediate/malignant tumors. ROC curve analysis was performed to evaluate the discriminatory ability of concave margins for certain types of lesions. Interobserver and intra-observer agreement for the number of concave margins was assessed using weighted Cohen’s kappa values, with kappa values interpreted as follows: 0–0.20 as slight; 0.21–0.40 as fair; 0.41–0.60 as moderate; 0.61–0.80 as substantial; and 0.81–1.00 as almost perfect agreement [18]. Quantitative Gaussian variables were expressed as mean and standard deviation (SD), while non-Gaussian quantitative variables were expressed as median, first quartile, and third quartile (Q1, Q3). The normality of data distribution was assessed graphically and using the Shapiro–Wilk test. *p*-values and Bonferroni-corrected *p*-values were considered significant at the 5% level. Statistical analyses were performed using R version 4.3.1 (R; R Foundation for Statistical Computing, Vienna, Austria).

## Results

The study group consisted of 586 lesions observed in 494 patients, with a median age of 46 years (range: 14 months to 86 years). Among these lesions, 296 (50.5%) were observed in males and 290 (49.5%) in females. These lesions were unique in 441 patients (89.3%), while 33 patients (6.7%) had two lesions, and 20 patients (4%) had between 3 to 7 lesions. The femur was the most frequently affected long bone, and the metaphysis was the most commonly involved region of the bone (Table 1). The study group included 440 (75.1%) tumors, of which 314 (53.6%) were benign and 126 (21.5%) were intermediate or malignant tumors, and 146 (24.9%) non-tumoral lesions (Table 2). The median lesion size was 3 cm (range: 1–44 cm).

**Table 1:** Bones affected by the lesions and localization in the bones

Long bone involved	
Femur	331 (56.5%)
Tibia	138 (23.5%)
Fibula	18 (3.1%)
Humerus	92 (15.7%)
Ulna	3 (0.5%)
Radius	4 (0.7%)
Localization in the long bone	
Epiphysis	47 (8%)
Metaphysis	194 (33.1%)
Diaphysis	119 (20.3%)
Epiphysis and metaphysis	59 (10.1%)
Metaphysis and diaphysis	151 (25.8%)
Epiphysis, metaphysis and diaphysis	16 (2.7%)

**Table 2:** List of lesions included in the study

	Type	Number of lesions
Tumoral benign lesions	Enchondroma	*N* = 132
	Non-ossifying fibroma	*N* = 79
	Fibrous dysplasia	*N* = 58
	Unicameral bone cyst	*N* = 23
	Aneurysmal bone cyst	*N* = 10
	Chondroblastoma	*N* = 7
Tumoral intermediate and malignant lesions	Myeloma/plasmacytoma	*N* = 53
	Metastasis	*N* = 34
	ACT and chondrosarcoma	*N* = 24
	Giant-cell tumor	*N* = 12
	Langerhans cell histiocytosis	*N* = 8
Non-tumoral lesions	Osteonecrosis	*N* = 71
	Mucoid cyst	*N* = 42
	Abscess	*N* = 24
	Brown tumor	*n* = 9

*ACT* atypical cartilaginous tumor

### Analysis

With each lesion having 6 borders, a total of 3516 borders were evaluated. The number of concave margins per lesion was significantly different between the lesion groups (*p* < 0.001). The effect size was large (η^2^ = 0.74). The number of concave margins per lesion, categorized by the nature of the lesion, is shown in Table 3. The percentage of concave margins per lesion, according to the type of lesion, is illustrated in the Electronic Supplementary Material section (Supplementary Fig. 1).

**Table 3:** Number of concave margins per lesion according to lesion type

Number of concave margins per lesion		0	At least 1	At least 2	At least 3	At least 4	At least 5	6
Tumoral benign lesions	Enchondroma (*n* = 132)	132 (100%)	0 (0%)	0 (0%)	0 (0%)	0 (0%)	0 (0%)	0 (0%)
	Non-ossifying fibroma (*n* = 79)	76 (96.2%)	3 (3.8%)	0 (0%)	0 (0%)	0 (0%)	0 (0%)	0 (0%)
	Fibrous dysplasia (*n* = 58)	20 (34.5%)	38 (65.5%)	26 (44.8%)	9 (15.5%)	4 (6.9%)	2 (3.4%)	0 (0%)
	Unicameral bone cyst (*n* = 23)	19 (82.6%)	4 (17.4%)	0 (0%)	0 (0%)	0 (0%)	0 (0%)	0 (0%)
	Aneurysmal bone cyst (*n* = 10)	7 (70%)	3 (30%)	0 (0%)	0 (0%)	0 (0%)	0 (0%)	0 (0%)
	Chondroblastoma (*n* = 7)	4 (57.1%)	3 (42.9%)	1 (14.3%)	0 (0%)	0 (0%)	0 (0%)	0 (0%)
Tumoral intermediate and malignant lesions	Myeloma/plasmacytoma (*n* = 53)	52 (98.1%)	1 (1.9%)	0 (0%)	0 (0%)	0 (0%)	0 (0%)	0 (0%)
	Metastasis (*n* = 34)	30 (88.2%)	4 (11.8%)	1 (2.9%)	0 (0%)	0 (0%)	0 (0%)	0 (0%)
	ACT/chondrosarcoma (*n* = 24)	23 (95.8%)	1 (4.2%)	0 (0%)	0 (0%)	0 (0%)	0 (0%)	0 (0%)
	Giant-cell tumor (*n* = 12)	12 (100%)	0 (0%)	0 (0%)	0 (0%)	0 (0%)	0 (0%)	0 (0%)
	Langerhans cell histiocytosis (*n* = 8)	6 (75%)	2 (25%)	1 (12.5%)	1 (12.5%)	0 (0%)	0 (0%)	0 (0%)
Non-tumoral lesions	Osteonecrosis (*n* = 71)	0 (0%)	71 (100%)	71 (100%)	71 (100%)	67 (94.4%)	54 (76.1%)	34 (47.9%)
	Mucoid cyst (*n* = 42)	42 (100%)	0 (0%)	0 (0%)	0 (0%)	0 (0%)	0 (0%)	0 (0%)
	Abscess (*n* = 24)	18 (75%)	6 (25%)	5 (20.8%)	2 (8.3%)	2 (8.3%)	1 (4.2%)	0 (0%)
	Brown tumor (*n* = 9)	9 (100%)	0 (0%)	0 (0%)	0 (0%)	0 (0%)	0 (0%)	0 (0%)

Percentage in bracket

*ACT* atypical cartilaginous tumor

**Fig. 2:**
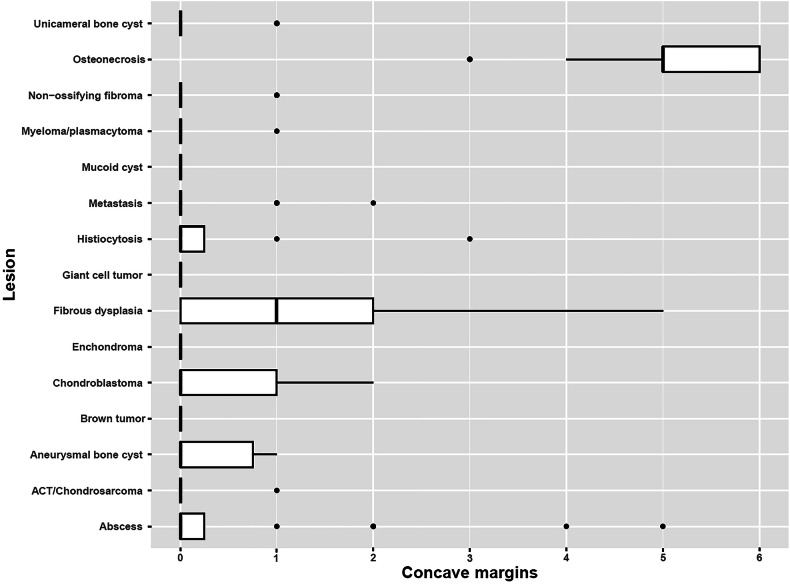
Box plot of the groups according to the number of concave margins in each lesion group. ACT, atypical cartilaginous tumor

A boxplot illustrating the number of concave margins for each lesion group is shown in Fig. 2. The post hoc analysis showed the following:

- The number of concave margins per lesion was significantly different between osteonecrosis from systemic origin and the other lesion groups (*p*_corrected_ < 0.01).
- The number of concave margins per lesion was significantly different between fibrous dysplasia on one side and the rest of the lesion groups, except for abscesses, chondroblastomas, Langerhans cell histiocytosis, and aneurysmal bone cyst (*p*_corrected_ < 0.01).
- There was no significant difference in the number of concave margins per lesion between the remaining pairs (*p*_corrected_ > 0.05).

**Fig. 3:**
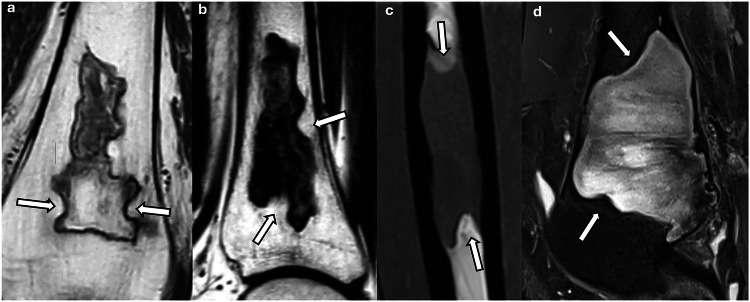
Lesions with at least two concave borders (arrows): **a**, **b** osteonecrosis; **c**, **d** fibrous dysplasia

**Fig. 4:**
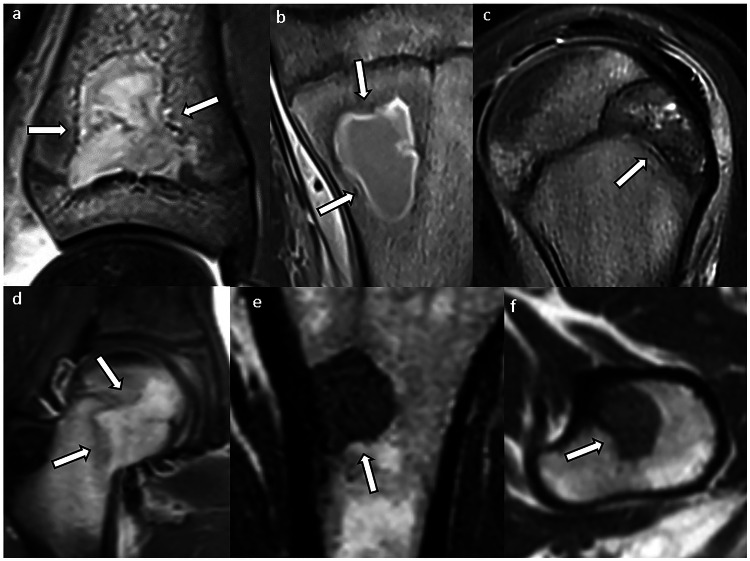
Lesions with concave borders (arrows): (**a**, **b**) intraosseous abscess, (**c**) chondroblastoma, (**d**) Langerhans cell histiocytosis, and (**e**, **f**) metastasis (same patient). In **a** and **c**, one concave border follows the shape of the growth plate

All 71 osteonecrosis lesions had at least two concave margins, followed by 26 fibrous dysplasia lesions (44.8%), five bone abscesses (20.8%), one chondroblastoma (14.3%), one Langerhans cell histiocytosis (12.5%), and one metastasis (2.9%) (Figs. 3 and  4). No enchondroma, chondrosarcoma, myeloma and plasmacytoma, non-ossifying fibroma, aneurysmal bone cyst, unicameral bone cyst, mucoid cyst, giant-cell tumor, or brown tumor had more than one concave margin.

### ROC curves

**Fig. 5:**
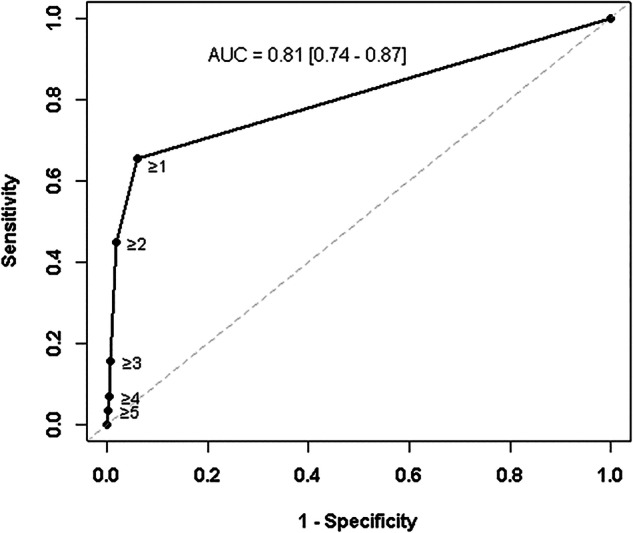
ROC curve assessing the ability of a concave margin presence to detect fibrous dysplasia. Each closed circle corresponds to concave margin number per lesion. The gray line is the chance line. The area under the curve (AUC) [95% confidence interval] is also indicated

**Fig. 6:**
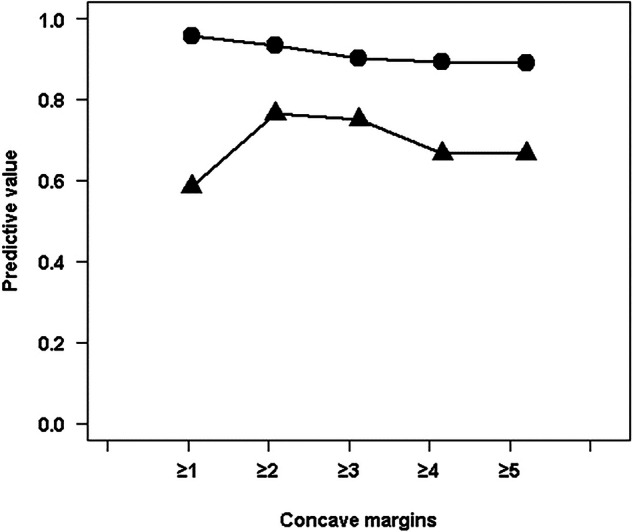
Positive (closed triangles) and negative (closed circles) predictive values of concave margin for the detection of fibrous dysplasia, as a function of the minimal concave margin number

ROC curve analysis was performed to assess the ability of the concave margins to discriminate between fibrous dysplasia and other bone lesions (Fig. 5). Osteonecrosis was excluded from this analysis. The AUC was 0.81 [0.74–0.87]. Predictive values were calculated (Fig. 6). The presence of two or more concave margins yielded a positive predictive value (PPV) of 0.76 when osteonecrosis was excluded.

### Lesion types analysis

Non-tumoral lesions had more frequent concave margins than tumoral lesions (*p* < 0.001). Benign tumors had more frequent concave margins than intermediate and malignant tumors (*p* = 0.0028). Benign lesions (non-tumoral lesions and benign tumors) had more frequent concave margins than intermediate and malignant tumors (*p* < 0.001). This difference remained statistically significant after excluding osteonecrosis (*p* = 0.0075).

The interobserver agreement for concave margins evaluation was estimated with a weighted Kappa value of 0.893; CI [0.87–0.92], and the intra-observer variability with a weighted Kappa value of 0.93; CI [0.89–0.97].

## Discussion

Incidental solitary bone lesions are frequently encountered on MRI in routine clinical practice. Accurate differentiation between benign and malignant bone tumors and non-tumoral processes is crucial for optimal patient management, which has led to the recent development of structured analysis of the lesions and guidelines [3, 9, 19]. While the aggressive nature of some tumors can be obvious on imaging, assessing this feature in purely intraosseous bone lesions may be more challenging.

This study was conducted based on the hypothesis that the presence of focal or extensive concave inward margins in a bone lesion may suggest non-aggressive behavior. To the best of our knowledge, the presence and value of this feature have not been fully described in the literature. Our study confirmed that both benign tumors and benign lesions (benign tumors and non-tumoral lesions) had more frequent concave margins than intermediate and malignant tumors. The comparison between non-tumoral lesions and benign or intermediate/malignant tumors was influenced by the large number of osteonecrosis cases.

Interestingly, six lesion groups exhibited at least two concave margins, whereas none of the lesions in the remaining groups showed more than one. The number of concave margins was associated with excellent intra- and interobserver agreements. However, it is important to differentiate true smooth inward concavities from pseudo-concavities related to indentations of the margins or lobulated margins, which are particularly common in cartilaginous tumors [6–8].

### Osteonecrosis from systemic origin

Systemic osteonecrosis was previously referred to as bone infarction when located in the metaphysis or diaphysis, but the term osteonecrosis is now recommended for all locations of devitalized bone [16, 20]. Osteonecrosis was the most common bone lesion in this study, with all cases exhibiting at least two concave margins. It was also the only condition in which every lesion displayed three or more concave margins. These concave outlines may correspond to the classic curvilinear or serpentine pattern well described in the literature [21, 22] and could reflect retraction of necrotic tissue [23–25]. As osteonecrosis usually shows typical features on MR imaging, including frequent fatty signal intensity, the identification of concave borders probably does not add much to the diagnosis of these lesions. Further studies could investigate whether the identification of concave margins is useful in cases with atypical imaging presentations (e.g., acute osteonecrosis, abnormal signal intensity of the adjacent bone marrow, etc.).

### Fibrous dysplasia

Fibrous dysplasia was the second most common lesion exhibiting concave margins in our study, with nearly half of the lesions having at least two concave margins. When osteonecrosis was excluded, the presence of two or more concave margins yielded a positive predictive value (PPV) of 0.76. This was one of the most striking results of our study, and, to the best of our knowledge, this feature has not been previously reported. This frequency of concave borders may seem surprising, as fibrous dysplasia is classified as a benign mesenchymal tumor in the 2020 WHO classification of bone tumors [26]. However, this lesion is still frequently considered a pseudotumor by radiologists because of its quiescent behavior or slow growth in most patients [27].

Fibrous dysplasia is composed of sparsely cellular fibrous stroma interlaced with curvilinear trabeculae of dysplastic woven bone. It typically enlarges slowly and indolently, allowing the surrounding bone time to remodel [27, 28]. Although we do not have a definitive explanation for the frequent occurrence of concave margins in fibrous dysplasia, two hypotheses may be proposed: (1) Imbalanced osteoclastic activity; GNAS mutation that characterizes FD disrupts osteoblastic function and upregulates cytokines—especially interleukin-6—that stimulate osteoclastic bone resorption. This resorption occurs both within the lesion and at its periphery [29, 30]. Superimposed on the lesion’s characteristically slow, centrifuge growth, such patchy osteoclastic activity could locally accentuate inward curvatures. (2) Mechanical explanation; woven bone in FD is structurally weaker than normal lamellar bone, while the surrounding normal bone undergoes remodeling in response to mechanical forces [31, 32]. Over time, areas of FD might become “compartmentalized” by regions of remodeled sclerotic bone or by irregular patterns of resorption.

The MR appearance of fibrous dysplasia, including its signal intensity and contrast enhancement, can be extremely variable [33], making its diagnosis with this imaging modality challenging, as shown in Fig. 3d, which was one of the cases that prompted our study. The depiction of at least two concave margins in lesions with non-fatty signal intensity might be useful for recognizing fibrous dysplasia and could be considered a reassuring feature.

### Intraosseous abscesses

Intraosseous abscesses showed at least two concave margins in one-fifth of cases. This percentage may seem surprising given the usual expansile nature of abscesses. However, abscesses are not always round or oval; they may also present a sinuous or serpentine appearance [34–36], which may account for the presence of concave margins. Interestingly, one lesion had a concave margin that followed the shape of the growth plate (Fig. 4a). However, this lesion also had four other concave margins that were distant from the growth plate. While the clinical and MR features of intraosseous abscesses are often straightforward, the presence of concave margins can be kept in mind in cases with unusual presentations (e.g., adults, chronic abscesses, etc.).

### Chondroblastomas

Only seven intraosseous chondroblastomas were included in our study; therefore, the conclusions regarding concave margins in this group of lesions remain limited. Only one lesion exhibited two concave margins, and two other lesions showed only one concave margin. Interestingly, three of these four concave margins could be attributed to an adjacent curved physis, which, at least temporarily, appeared to restrain the growth of the chondroblastoma. Therefore, the presence of concave margins in chondroblastomas seems mainly related to their epiphyseal location and their perilesional environment, particularly when these benign cartilaginous tumors are detected early and/or exhibit mild aggressiveness.

### Langerhans cell histiocytosis

Only one lesion out of eight demonstrated two concave margins. Although the small sample size limits the conclusions, this sign should be kept in mind, as this disorder can mimic aggressive tumoral processes, particularly in children and adolescents.

### Metastasis

Finally, only one metastasis out of 34 showed two concave borders, which may appear surprising given the usual expansile behavior of such tumoral lesions. This exception could be explained by the lesion’s small size (15 mm) and its relatively indolent behavior due to the underlying neuroendocrine renal primary tumor. However, larger studies are needed to confirm the rarity of this feature in metastases.

### Limitations

We acknowledge several limitations in our study. First, this was a single-center study, which influenced the nature of the lesions assessed on MRI, even though both tumor and non-tumor lesions were included. Second, only 586 lesions were included in our study. Larger multicenter studies might demonstrate different prevalences of concave margins in the lesions we studied, as well as other causes of lesions with concave borders. Third, the limited number of several types of lesions contrasted with the large group of osteonecrosis. This is mainly due to the higher frequency of osteonecrosis and the exclusion of lesions with cortical destruction, which prevents the analysis of bone margins. However, the aggressive nature of such lesions is typically evident on imaging, likely rendering the identification of concave margins unnecessary. Fourth, our investigation was confined to long bones; therefore, its applicability to flat or small tubular bones—such as the calvarium, ribs, or phalanges—remains uncertain. In these locations, the medullary cavity is narrow, making lesion margins difficult to delineate on MRI, with many lesions abutting the cortex. Moreover, we did not evaluate facial bones in this study, as this anatomical region is assessed by neuroradiologists at our institution. Further studies are therefore needed to determine whether concave margins might also be applicable to skeletal sites other than long bones.

## Conclusion

In conclusion, our study demonstrated the propensity of several intraosseous lesions to present concave margins. When assessing an intraosseous lesion on MRI, noting at least two smooth inward-curving margins may help, as this sign seems to occur predominantly in benign lesions. The depiction of this feature in non-fatty lesions may be useful for recognizing fibrous dysplasia. Larger multicenter studies might be undertaken to confirm this information. Multicenter studies now need to be conducted to confirm the usefulness of this sign in clinical practice and to determine its incremental value over established imaging criteria.

## Supplementary information

ELECTRONIC SUPPLEMENTARY MATERIAL

## Data Availability

Availability of data and materials is available upon demand. Data and material are stored in a safe environment on Lille University Hospital servers, without possibility of external access, as requested by the Hospital Institution’s Data Protection Officer. Separated files of the dataset can be sent if necessary, upon the Institution’s Data Protection Officer’s approval.

## Abbreviations

ACT: Atypical cartilaginous tumor
FD: Fibrous dysplasia
MRI: Magnetic resonance imaging
