# Supplementary material for: Concavities of the margins of focal bone lesions on MRI: a retrospective study of 586 cases
Source: Insights Imaging. 2025 Nov 10;16:251. doi: 10.1186/s13244-025-02137-9 (PMC12602811; doi:10.1186/s13244-025-02137-9)
Supplement: Supplementary file 1 — ELECTRONIC SUPPLEMENTARY MATERIAL [file 13244_2025_2137_MOESM1_ESM.pdf]

Concavities of the margins of focal bone lesions on MRI, a retrospective study of 586 cases

ELECTRONIC SUPPLEMENTARY MATERIAL

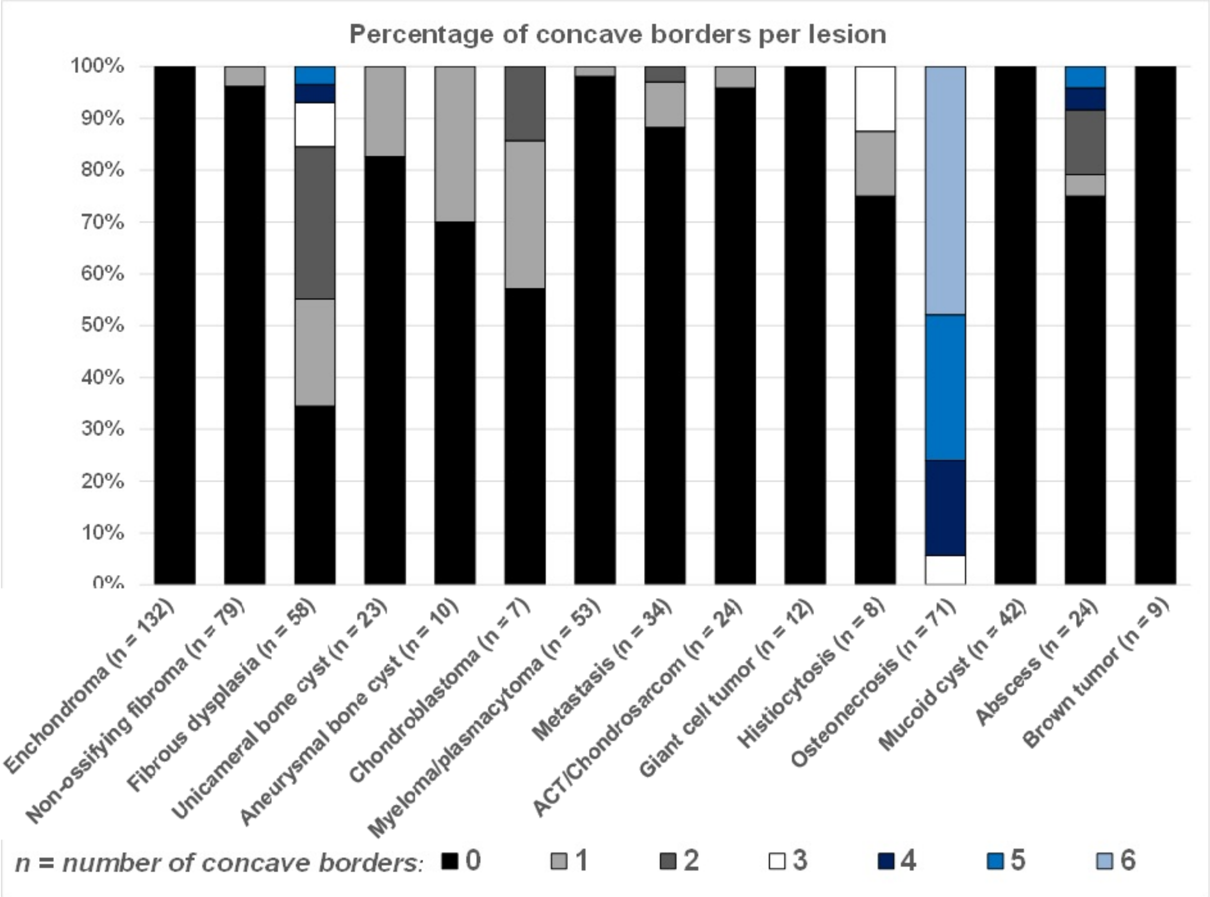

Supplementary figure 1: Percentage of concave margins per lesion - osteonecrosis
